# Glycemic Index, Glycemic Load and Cancer Risk: An Updated Meta-Analysis

**DOI:** 10.3390/nu11102342

**Published:** 2019-10-02

**Authors:** Federica Turati, Carlotta Galeone, Livia S. A. Augustin, Carlo La Vecchia

**Affiliations:** 1 Department of Clinical Sciences and Community Health, Università degli Studi di Milano, 20133 Milan, Italy; federica.turati@unimi.it (F.T.); carlotta.galeone@gmail.com (C.G.); 2 Clinical Nutrition and Risk Factor Modification Centre, St Michael’s Hospital, Toronto, ON M5B 1W8, Canada; livia.augustin@utoronto.ca; 3 National Cancer Institute, SSD di Epidemiologia, Istituto Nazionale Tumori - IRCCS - “Fondazione G. Pascale”, 80131 Naples, Italy

**Keywords:** cancer, glycemic index, glycemic load, review, risk

## Abstract

Diets high in glycemic index (GI) and glycemic load (GL) have been related to an increased risk of selected cancers, but additional quantification is required. We updated a systematic review and meta-analysis published in 2015 to May 2019 to provide quantitative information on GI/GL and cancer risk. Relative risks (RR) and the corresponding 95 % confidence intervals (CI) for the highest versus the lowest categories of GI and GL were extracted from selected studies and pooled using random-effects models. Twenty reports (>22,000 cancer cases) have become available after January 2015, and 15 were added to the meta-analyses by cancer sites, which considered a total of 88 investigations. The five additional reports were reviewed, but not included in the meta-analyses, since data were inadequate to be pooled. For hormone-related cancers, summary RRs for the highest versus lowest GI and GL intakes were moderately increased. They ranged from 1.04 (breast) to 1.12 (endometrium) for GI and from 1.03 (prostate) to 1.22 (ovary) for GL, of borderline significance. High GI was associated with small increased risks of colorectal (summary RR for GI: 1.20, 95% CI, 1.07–1.34—GL: 1.09, 95% CI, 0.97–1.22, 19 studies), bladder (GI: 1.25, 95% CI, 1.11–1.41—GL: 1.10, 95% CI, 0.85–1.42, four studies) and kidney cancers (GI: 1.16, 95% CI, 1.02–1.32—GL: 1.14, 95% CI, 0.81–1.60, five studies). GL was not significantly related to those cancer sites. Stomach, prostate and lung cancers were not associated with GI and GL. The present analysis, based on an updated comprehensive evaluation of the epidemiological literature, indicates moderate unfavorable effects of high versus low GI on colorectal, and possibly bladder and kidney cancers, and a possible moderate positive association between GL and endometrial cancer.

## 1. Introduction

The glycemic index (GI) is an index of carbohydrate foods which indicates how quickly the food causes an increase in blood glucose levels [1]. High GI foods have fast-release carbohydrates and higher blood glucose concentrations, leading to increased insulin secretion. Low GI foods are digested, absorbed and metabolized more slowly, thus, resulting in a more gradual rise in blood glucose. The glycemic load (GL) is the product of GI and the total available carbohydrate content in a given amount of food. It reflects both the quality (i.e., GI) and the quantity of carbohydrates [2].

Postprandial glycaemia, and consequently dietary GI and GL have been related to diabetes, coronary heart disease (CHD) and obesity [3,4,5]. 

A possible role in the development of selected cancers has been also suggested [6], but the evidence is yet weak [7]. Long-term consumption of a high GI/GL diet results in chronically high blood glucose, and hence, to chronically elevated insulin concentration. Insulin increases bioactive IGF-1, which promote cancer development by inhibiting apoptosis and stimulating cell proliferation [8,9]. In addition, hyperglycemia, insulin resistance, diabetes and obesity, which are linked to glucose metabolism, may affect the risk of cancer [4,10,11,12]. 

In 2015, we systematically reviewed data from 75 epidemiological reports addressing the association between dietary GI and GL and the risk of cancer, for over 147,000 cancer cases [13]. Seventy-two studies were included in the meta-analyses by cancer sites. The summary relative risks (RR) of hormone-related cancers for the highest versus the lowest study-specific category ranged from 1.05 (95% confidence interval, CI, 0.99–1.11, breast cancer) to 1.13 (95% CI, 0.98–1.32, endometrial cancer) for GI, and from 1.04 (95% CI, 0.91–1.18, prostate cancer) to 1.19 (95% CI, 0.85–1.68, ovarian cancer) for GL. A significantly increased colorectal cancer risk emerged for high GI (summary RR = 1.16, 95% CI, 1.07–1.25). Other summary RRs were not significantly above unity.

After that review, 20 reports [14,15,16,17,18,19,20,21,22,23,24,25,26,27,28,29,30,31,32,33] have been published, accounting for 23 distinct original studies and almost 22,000 additional cancer cases. These included the EPIC-Italy cohort investigating multiple cancer sites [26], the Framingham Offspring cohort on adiposity-related cancers [29], a pooled analysis of two studies on esophageal and gastric cardia adenocarcinoma [24], three studies on colorectal cancer [17,30,31], one combined analysis of two North European cohorts on cancer of the biliary tract [19], two studies on lung cancer [22,25] (including a combined analysis of two Asian cohorts [25]), one on melanoma [23], four on breast cancer [14,15,16,32] (including the update to 2011 of the Nurses’ Health Study II (NHSII) [16]), two on endometrial cancer [20,33], one on ovarian cancer [21], one on bladder cancer [28], one on kidney cancer [27], and one on thyroid cancer [18]. 

A few meta-analyses have recently reported on GI/GL and a single cancer [34,35,36,37,38,39], but no study has provided an updated comprehensive quantification of the association of GI/GL with a wide range of cancer types in a single report. 

Given the new original data, we updated our systematic review and meta-analysis [13] to derive a more precise estimation of the associations. Specifically, we reviewed the most recent (up to May 2019) epidemiological data on the association between dietary GI and GL and cancer risk, updated summary RRs for stomach, colorectal, pancreatic, breast, endometrial, ovarian and prostate cancers and addressed issues of heterogeneity and complexities of interpretation [40]. Moreover, we calculated meta-analytic estimates for kidney, and for the first time, lung and bladder neoplasms.

## 2. Materials and Methods

### 2.1. Search Strategy and Selection Criteria

We conducted a systematic review and a series of meta-analyses on dietary GI and dietary GL and cancer risk following recognized reporting guidelines [41]. We included relevant studies selected in our prior systematic review and meta-analysis on GI/GL intake and cancer risk (search up to January 2015 [13]), and updated the search using the same search strategy through May 2019. Briefly, the following string was used in Medline/Pubmed, with no language or other restriction: “((cancer) OR (neoplasm) OR (carcinoma)) AND ((glycemic index) OR (glycemic load) OR (glycaemic index) OR (glycaemic load))”. The electronic search was supplemented by hand searching of references of the selected publications and previous reviews.

We selected original cohort or case-control studies that assessed the association between dietary GI and/or GL intake and the incidence of or mortality from a specific cancer (Table 1). Studies analyzing cancer overall were excluded [42]. To be included in the quantitative meta-analyses, studies had to report the estimates of the RRs (i.e., odds ratios, ORs, or RRs, or hazard ratios, HRs) and the corresponding 95% CI for categories of GI/GL intake, or information needed for their calculation; studies providing only the RR for the increment of one unit of GI/GL were reviewed, but not considered in the meta-analyses [32,43]. We conducted meta-analyses for cancer sites investigated in at least four studies.

Two reviewers independently identified eligible studies by screening titles, abstract, and when appropriate, full-texts of articles. Any discrepancy was discussed and resolved by consensus. If there were multiple publications from the same study population, we used data from the longest follow-up (for cohorts) or the larger database (for case-control studies).

From each selected publication, we extracted details on study design, country, sex of the participants, tumor site, number of enrolled subjects (cases and controls or cohort size), period of enrolment (for case-control studies) or follow-up (for cohort studies), methods for dietary assessment (type, number of food items, and whether it had been validated), reference food for GI/GL (white bread or glucose), comparison levels of GI and/or GL, RR estimates (i.e., odds ratios/hazard ratios/RRs) for the comparison between the highest versus the lowest category of GI/GL intake, and confounding factors for which adjustment was made. When RR estimates from models with different covariates were provided, we extracted the RRs adjusted for the largest number of confounding factors. 

### 2.2. Statistical Analysis

We conducted quantitative meta-analyses of GI and GL intake in association with the risk of cancer. In the current analysis, we considered cancer sites investigated in at least four studies and for which new epidemiological data have become available after the previous review [13], i.e., breast, endometrium, ovary and prostate among hormone-related cancers, stomach, colorectum and pancreas among digestive tract cancers, and lung, bladder and kidney among other cancer sites. Although new data on liver cancer have been made available from the EPIC-Italy cohort [26], we did not update the meta-analysis on liver cancer as that data partially overlapped with those reported in 2013 by Fedirko et al. [44], which analyzed the overall EPIC cohort. 

We derived summary estimates of the RR by combining study-specific ORs or RRs or HRs for the highest versus the lowest category of GI and GL intake using random-effects models, which account for the heterogeneity among the RR estimates. Heterogeneity was evaluated through the χ^2^ test and quantified through and the *I*^2^ statistic [45,46]. 

We updated subgroup analyses for breast cancer according to menopausal status and body mass index (BMI) (<25 kg/m^2^ and BMI ≥ 25 kg/m^2^). One study showing the RR of breast cancer according to GI and GL among women with BMI ≥ 30 kg/m^2^ was excluded from the subgroup analysis [14].

Publication bias was assessed through funnel plots and Egger tests [47]. 

All the statistical analyses were performed using the STATA software (version 14; StataCorp, College Station, TX, USA).

## 3. Results

Of the 203 newly identified publications in the Medline literature (from January 2015 to May 2019), 20 were selected as original studies providing results on the association between GI and/or GL and the risk of a specific cancer (Table 1) (for a detailed description of studies on GI/GL and cancer risk published before January 2015, refer to Table 1—Supporting information of our previous work [13]). Eleven reports provided results from case-control studies (including one report of a pooled analysis of two case-control studies [24]) and nine from cohort studies (including a combined analysis of two cohorts in Shanghai [25], a combined analysis of two Swedish cohorts [19], and a prospective analysis based on data from the PREvención con DIeta MEDiterránea (PREDIMED trial) [14]). Two reports gave results on a number of cancer sites [26,29]; the remaining reports were focused on a single cancer type. These reports included a minimum of 32 [14] to a maximum of 5112 cases [26]. Eight reports were based on studies conducted in North America, three in Central/South America, six in Europe, and three in Asia. All studies except one [20] provided results for both GI and GL. Validated FFQs were used in all studies to evaluate dietary habits. Most studies used glucose as reference food for GI/GL calculation. GI/GL were categorized in quartiles in 11 reports, in quintiles in five reports and in tertiles in three reports; one study used GI/GL as a continuous variable.

Fifteen of the 20 newly identified reports provided data that could be added to our meta-analyses on GI/GL and cancer risk. One publication on breast cancer providing only the RRs for the increment of one unit of GI/GL [32], and four publications on cancers sites for which less than four studies were available (i.e., esophagus and gastric cardia [24], biliary tract [19], melanoma [23], thyroid [18]), were not considered in the quantitative meta-analyses. In the meta-analyses on breast cancer, the 2015 paper by Farvid et al. [16] with updated results of the NHSII cohort replaced the 2003 paper by Cho et al. [48], which was based on a shorter follow-up and a quarter of the cases. Similarly, in the meta-analyses on colorectal cancer, updated results from the EPIC-Italy cohort [26] replaced data from an earlier publication on the same cohort [49]. Updated data from the EPIC-Italy cohort [26] did not contribute to the meta-analyses on breast and endometrial cancers as the analyses already included larger amounts of data from the overall EPIC cohort [50,51]. In addition, we retrieved one publication on ovarian cancer not previously identified [52]. 

### 3.1. Results of the Individual Studies

#### 3.1.1. Multiple Cancer Sites

The EPIC-Italy cohort provided results for 21 different cancer types [26]. In a median follow-up of about 15 years, 5112 incident cancers occurred (including 441 colon, 102 rectal, 117 pancreatic, 307 lung, 1362 breast, 203 endometrial, 135 ovarian, 481 prostate, 251 bladder and 136 kidney cancers). For most cancer sites, no association was found for either dietary GI or GL. High GI was associated with an increased risk of colon (HR for the fifth versus first quintile: 1.48, 95% CI, 1.09–2.01) and bladder cancer (HR: 1.51, 95% CI, 1.01–2.25), and high GL with an increased risk of colon (HR: 1.80, 95% CI, 1.18-2.74) and diabetes-related cancers (HR: 1.23, 95% CI, 1.03–1.48), but a decreased risk of rectal cancer (HR: 0.42, 95% CI, 0.18–0.98). The Framingham Offspring cohort found no association between GI, GL and adiposity-related cancers, based on a total of 656 incident cancers of the gastrointestinal tract, reticuloendothelial system (blood, bone and spleen), female reproductive tracts, genitourinary organs and the thyroid gland [29]. In analyses by cancer type, no significant associations were found for breast, colorectal, and prostate cancers. The HRs for the third versus first tertile were 0.90 (95% CI, 0.59–1.37) for GI and 0.54 (95% CI, 0.26–1.09) for GL for breast cancer (124 cases), 1.51 (95% CI, 0.81–2.84) for GI and 1.21 (95% CI, 0.43–3.40) for GL for colorectal cancer (68 cases), and 0.74 (95% CI, 0.48–1.12) for GI and 0.76 (95% CI, 0.40–1.43) for GL for prostate cancer (157 cases). 

#### 3.1.2. Hormone-Related Cancers

As for breast cancer, the update of the NHSII, which included 12 additional years of follow-up and almost four times the number of cases compared to the 2003 report [48], found a null association between GI and GL and breast cancer risk, with HRs for the fifth versus the first quintile of 1.03 (95% CI, 0.91–1.16) for GI and 0.94 (95% CI, 0.83–1.06) for GL, based on 2833 invasive cases [16]. Similar null results were observed in pre- (HR for GI: 1.05, HR for GL: 0.93) and post-menopause (HR for GI: 1.08, HR for GL: 0.95), as well as among normo- (HR for GI: 1.04, HR for GL: 0.94) and over-weight women (HR for GI: 1.12, HR for GL: 1.19, not significant). In a secondary analysis within the framework of the PREDIMED trial, based on 32 incident cases, dietary GI and GL were not related to breast cancer among postmenopausal women at high risk of cardiovascular diseases (HR for the third versus the first tertile 1.02, 95% CI, 0.42–2.46 for GI and 1.00, 95% CI, 0.44–2.30 for GL) [14]. No association with breast cancer was also reported in a Mexican population-based case-control study including 1000 women with breast cancer, the ORs for the fourth versus the first quartile being 0.90 (95% CI, 0.68–1.2) for GI and 1.1 (95% CI, 0.82–1.1) for GL [15]. In that study, a null association was also observed in subgroups defined by menopausal status and BMI. A further Mexican case-control study, including 509 cases matched 1:1 by age with 509 population controls, found a positive association between GI and breast cancer overall (OR for 1 unit increase 1.15, 95% CI, 1.09–1.23) and luminal A, HER2+ and TN molecular subtypes; no association emerged for GL regardless of the subtype [32]. 

Two studies investigating GI/GL in relation to endometrial cancer found both an absence of association [20,33]. The HRs were 0.98 (95% CI, 0.74–1.29) for GI and 0.83 (95% CI, 0.62–1.11) for GL for the fourth versus first quartile in the US Cancer Prevention Study II (CPS-II) Nutrition Cohort, which included 425 incident cases occurred during a median follow-up of about 14 years [33]. In addition, a Canadian case-control study with over 500 cases and 980 population controls found an OR of 0.87 (95% CI, 0.52–1.49) for the fourth versus the first quartile of dietary GL, with no effect modification by BMI [20].

As for ovarian cancer, a case-control study among African-Americans in the USA enrolling about 400 cases and 600 population controls found no association with GI (OR for the fourth versus the first quartile 1.03, 95% CI, 0.84–1.18), but a suggestion of a positive association with GL [21]. The ORs were 1.18 (95% CI, 1.04–1.33) per 10 units of GL/1000 kcal in the continuous analysis and 1.35 (95% CI, 0.93–1.97) for the highest versus the lowest quartile of GL when the variable was considered in categories; in the latter analysis, however, a clear dose-risk relationship did not emerge (ORs for subsequent quartiles: 1.16, 1.57 [significant], 1.35, p for trend = 0.05) [21]. 

#### 3.1.3. Cancers of the Digestive Tract

Two case-control [30,31] and one cohort study [17] on colorectal cancer have become available after the previous meta-analysis. The Japan Public Health Center-based prospective Study (JPHC Study) provided, for the first time, data on GI/GL and colorectal cancer risk in a Japanese population [17]. Based on 1468 incident cases from a population of over 73,000 subjects followed for 12.5 years on average, the JPHC study found overall non-significant results for GI, as well as GL; the HRs of colorectal cancer for the fourth versus the first quartile were 0.92 (95% CI, 0.73–1.14) in men and 0.97 (95% CI, 0.73–1.30) in women for GI, and 0.79 (95% CI, 0.58-1.08) in men and 0.82 (95% CI, 0.55–1.24) in women for GL. However, a non-significantly reduced risk of (proximal) colon cancer for high GL consumption was observed in men (HRs for proximal colon cancer: 0.62, 95% CI, 0.36–1.08) and a non-significantly reduced risk of rectal cancer for high GI (HRs 0.58, 95% CI, 0.33–1.03) and GL (HR: 0.52, 95% CI, 0.24–1.14) was found in women [17]. A Chinese case-control study, including 1944 cases and 2027 community-derived or hospital controls, reported a positive association between GI and colorectal (OR for the fourth versus first quartile: 3.10, 95% CI, 2.51–3.85), as well as colon (1172 cases) and rectal cancer (772 cases), similar in men and women [31]. High GL was not associated with colorectal (OR for the fourth versus first quartile: 1.14, 95% CI, 0.94–1.35), colon or rectal cancer. However, an increased risk of colorectal cancer for the highest versus lowest quartile of GL was found in women only (OR 1.42, 95% CI, 1.04–1.95). A case-control study from Argentina and based on 161 cases of colorectal cancer and 331 population-based controls showed significant positive associations with GI and GL in women only (OR for the third versus first tertile 2.12, 95% CI, 1.38–3.27 for GI, OR: 1.98, 95% CI, 1.24–3.18 for GL) [30]. 

#### 3.1.4. Other Neoplasms

A pooled analysis of two US population-based case-control studies (i.e., the US Multi-Center Study and the Los Angeles Multi-Ethnic Study), including overall 500 esophageal adenocarcinomas, 529 gastric cardia adenocarcinomas and 2027 controls, reported an increased risk of esophageal adenocarcinoma for the fifth versus the first quintile of GI intake (OR 1.58, 95% CI, 1.13–2.21), in the absence, however, of a clear dose-risk relationship (p for trend 0.32). Such positive association was evident only among subjects with BMI ≥ 25 kg/m^2^. No relation emerged between GI and esophageal adenocarcinoma (OR for the fifth versus the first quintile: 1.21, 95% CI, 0.88–1.67), as well as between GL intake and esophageal (OR: 0.81, 95% CI, 0.51–1.29) and gastric cardia adenocarcinoma (OR: 0.86, 95% CI, 0.55–1.35) [24]. 

Dietary GI and GL were positively related to the risk of biliary tract cancer in the combined analysis of the Swedish Mammography Cohort and cohort of Swedish Men, which included 140 extrahepatic and 23 intrahepatic cancer cases developed from a population of over 76,000 individuals during a mean follow-up of 13.3 years [19]. The HRs for the fourth versus first quartile of dietary GI was 2.12 (95% CI, 1.25–3.58) for extrahepatic and 1.47 (95% CI, 0.54–3.97) for intrahepatic biliary tract cancer; the corresponding values for GL were, respectively, 1.63 (95% CI, 1.01–2.63) and 3.46 (95% CI, 1.22–9.84) [19].

Two reports gave results on GI/GL and lung cancer [22,25]. Data from the Shanghai Women’s and Men’s Health Studies, including 649 incident lung cancers among women and 663 among men developed during an average follow-up of about 15 years, indicated no association of GI or GL with lung cancer [25]. The HRs for the fourth versus the first quartile of GI was 1.16 (95% CI, 0.92–1.47) in women and 0.83 (95% CI, 0.67–1.03) in men; the corresponding figures for GL were 1.09 (95% CI, 0.86-1.37) in women and 0.85 (95% CI, 0.68–1.05) in men. Null findings were also found in strata of BMI, smoking, family history of cancer and menopausal status (in women). In a population-based case-control study from USA based on 1905 incident cases and 2413 controls [22] a significant positive association with lung cancer risk was found for GI (OR for the fifth versus the first quintile 1.49, 95% CI, 1.21–1.83, *p* for trend < 0.001), but not for GL (OR 1.16, 95% CI, 0.94–1.42). The positive association with GI was more evident among never smokers (OR fifth quintile 2.25, 95% CI, 1.42–3.57) and for the squamous cell carcinoma histologic subtype (OR 1.30, 95% CI, 1.02–1.67) [22]. 

An Italian population-based case-control study including 380 cases of melanoma and 719 age- and sex-matched controls reported a positive association with GL among women (OR 2.38; 95% CI 1.25, 4.52 for the highest versus the lowest quintile, *p* for trend = 0.070), but not among men (OR 0.86, 95% CI, 0.47–1.57), and a lack of relation with GI in either sex [23]. 

A positive association between GL and bladder cancer risk was found in a case-control study from Italy, including 578 cases and 608 hospital-controls. The risk of bladder cancer was significantly increased from the second quartile onwards, and subjects in the fourth quartile had an approximately doubled risk of bladder cancer compared to those in the first one (OR 1.96, 95% CI, 1.16–3.31) [28]. No association emerged with GI. 

One case-control study from USA investigated renal cell carcinoma and found a positive association for GI and an absence of association for GL [27]. Based on a total of 854 cases and 1255 population-based controls, the ORs for the fourth versus the first quartile were 1.32 (95% CI, 0.99–1.74; *p* for trend = 0.026) for GI and 1.15 (95% CI, 0.88–1.51) for GL.

An analysis based on 556 well-differentiated thyroid cancers within the EPIC cohort found overall no association with GI (HR for the fourth versus first quartile: 0.94, 95% CI, 0.73–1.20) and GL (HR: 0.95, 95% CI, 0.74–1.24) [18]. In stratified analysis, a significant positive relation among overweight (HR: 1.54, 95% CI, 1.05–2.28, *p* for trend = 0.014) and an inverse one among normoweight subjects (HR: 0.64, 95% CI, 0.46–0.89, *p* for trend = 0.003) emerged with GI [18]. 

### 3.2. Update of the Summary Estimates

**Figure 1:**
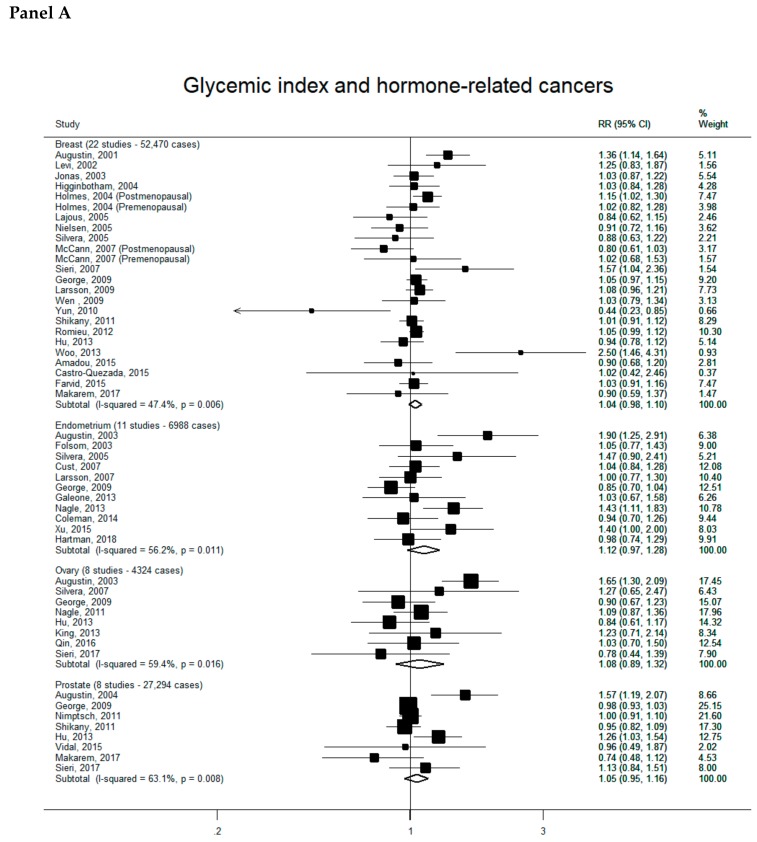

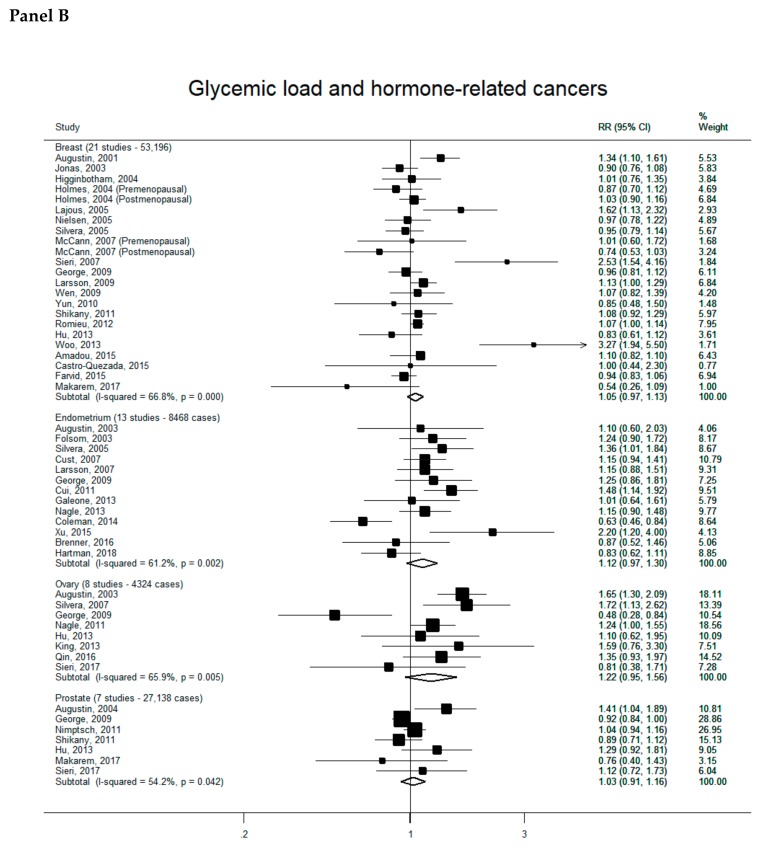
Study-specific and summary relative risks (RRs) of hormone-related cancers for the highest versus the lowest category of glycemic index (Panel A) or glycemic load intake (Panel B).

**Figure 2:**
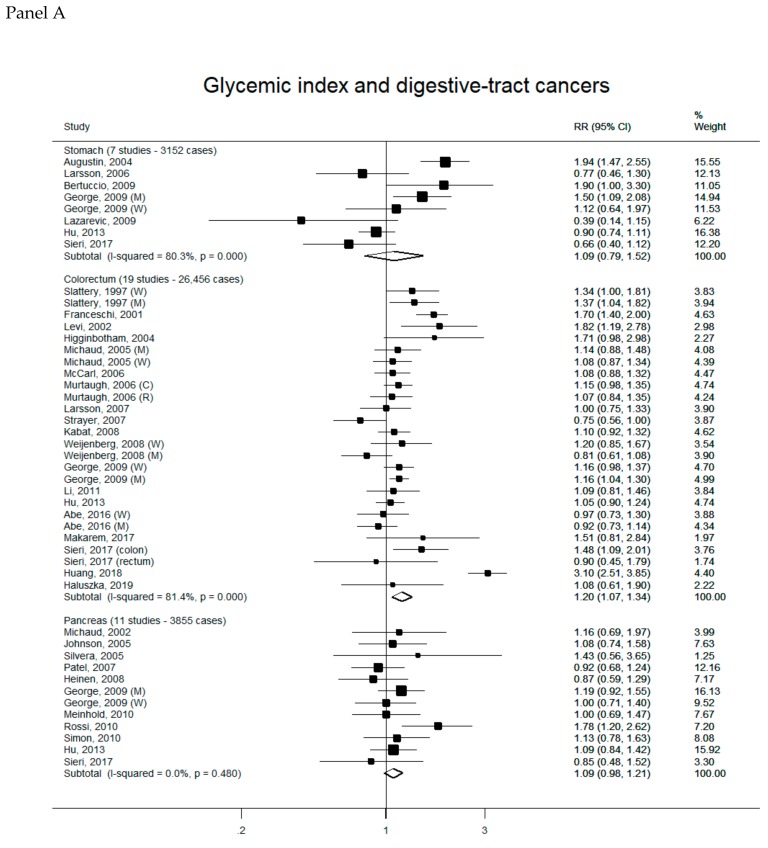

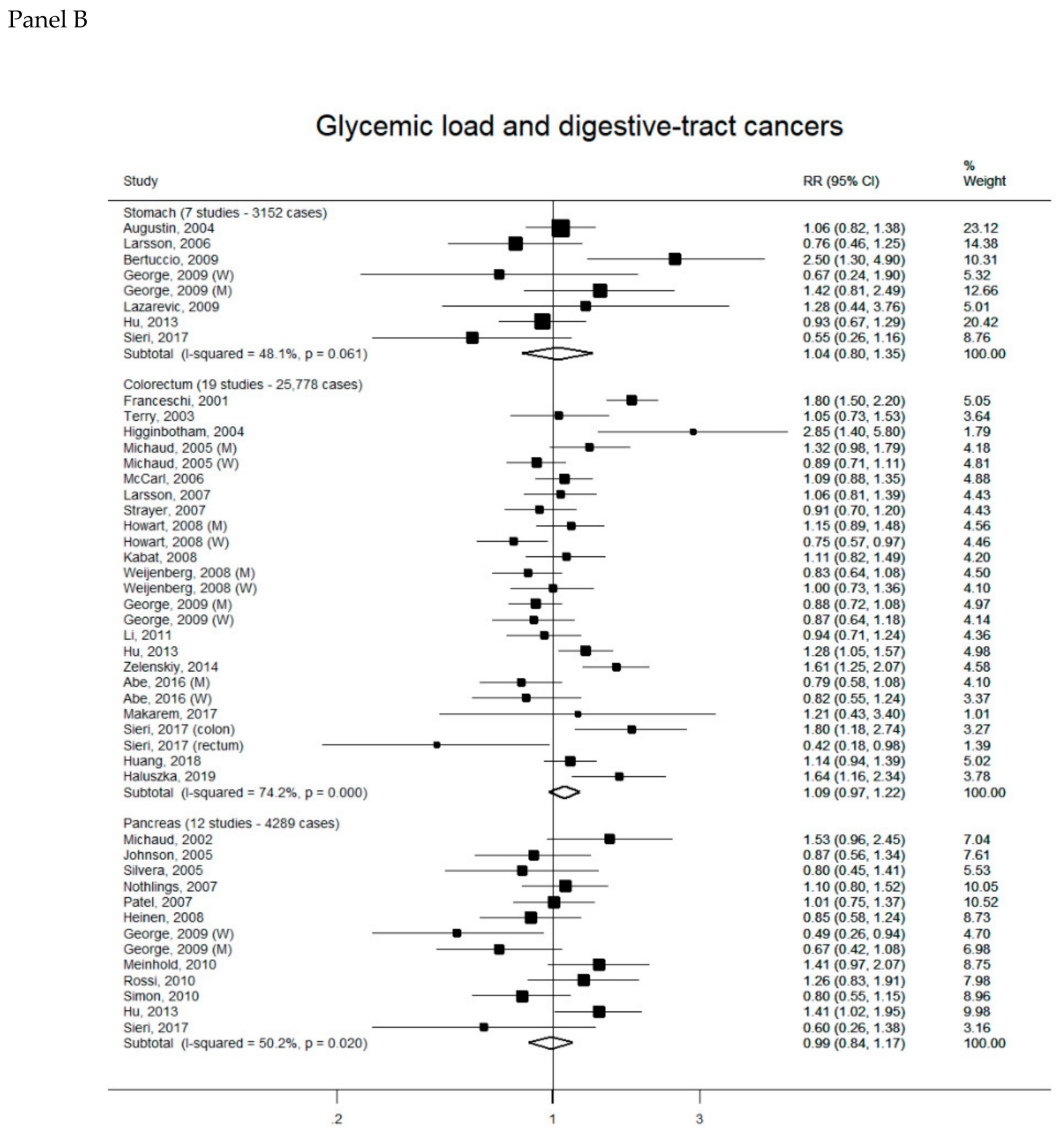
Study-specific and summary relative risks (RRs) of digestive-tract cancers for the highest versus the lowest category of glycemic index (Panel A) or glycemic load intake (Panel B).

**Figure 3:**
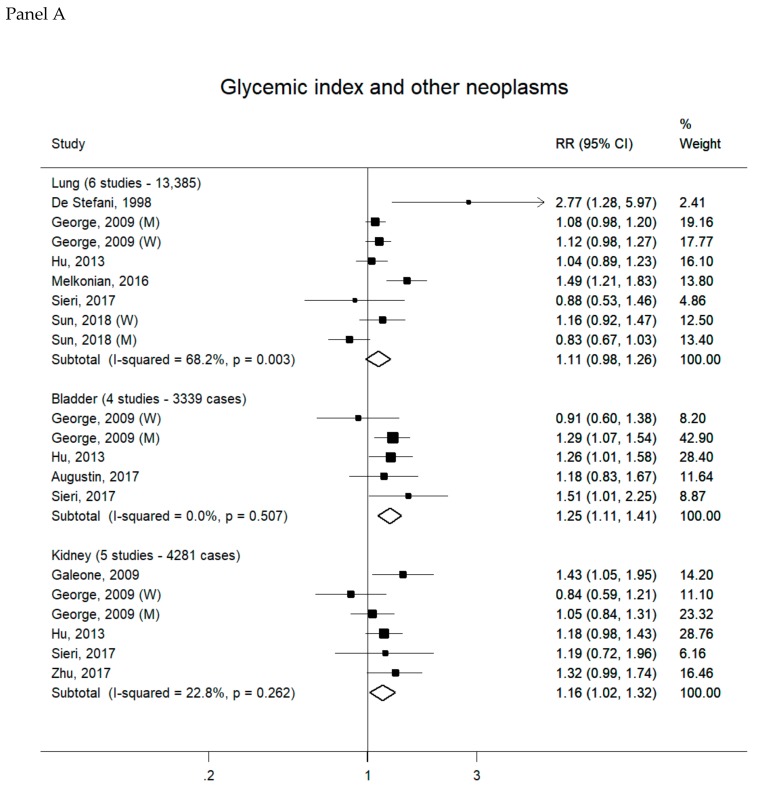

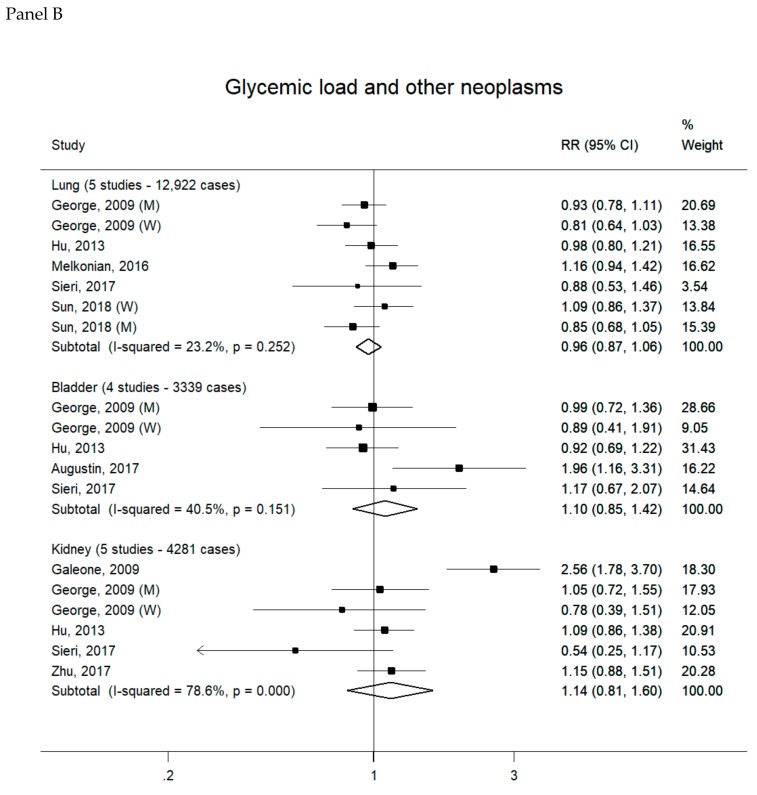
Study-specific and summary relative risks (RRs) of other neoplasms for the highest versus the lowest category of glycemic index (Panel A) or glycemic load intake (Panel B).

Overall, 88 reports were included in the meta-analyses on GI/GL and cancer risk (see Supplementary Materials for complete bibliography references). Summary RRs of hormone-related cancers, digestive-tract cancers and other neoplasms for the highest versus the lowest category of GI (Panel A) and GL (Panel B) intake are presented, respectively, in Figure 1, Figure 2 and Figure 3. There was no evidence of publication bias according to the Egger’s tests for all cancer sites considered (data not shown). 

#### 3.2.1. Hormone-Related Cancers

Summary RR of hormone-related cancers were all above unity, but non-significant, for both GI and GL, for all cancer sites (Figure 1). They were 1.04 (95% CI, 0.98–1.10) for GI (22 studies, 52,470 cases-Panel A) and 1.05 (95% CI, 0.97–1.13) for GL (21 studies, 53,196 cases-Panel B) for breast cancer, 1.12 (95% CI, 0.97–1.28) for GI (11 studies, 6988 cases) and 1.12 (95% CI, 0.97–1.30) for GL (13 studies, 8468 cases) for endometrial cancer, 1.08 (95% CI, 0.89–1.32) for GI (eight studies, 4324 cases) and 1.22 (95% CI, 0.95–1.56) for GL (eight studies, 4324 cases) for ovarian cancer, and 1.05 (95% CI, 0.95–1.16) for GI (eight studies, 27,294 cases) and 1.03 (95% CI, 0.91–1.16) for GL (seven studies, 27,138 cases) for prostate cancer. Significant heterogeneity across studies was observed in all the meta-analyses. 

#### 3.2.2. Digestive-Tract Cancers

There was a positive significant association between high GI intake and colorectal cancer risk. No other significant associations were detected (Figure 2). Summary RRs were 1.09 (95% CI, 0.79-1.52) for GI (seven studies, 3152 cases—Panel A) and 1.04 (95% CI, 0.80–1.35) for GL (seven studies, 3152 cases–Panel B) for stomach cancer, 1.20 (95% CI, 1.07–1.34) for GI (19 studies, 26,456 cases) and 1.09 (95% CI, 0.97–1.22) for GL (19 studies, 25,778 cases) for colorectal cancer, 1.09 (95% CI, 0.98–1.21) for GI (11 studies, 3855 cases) and 0.99 (95% CI, 0.84–1.17) for GL (12 studies, 4289 cases) for pancreatic cancer. Significant heterogeneity across studies was detected in all the meta-analyses, except for that on GI and pancreatic cancer. In a sensitivity analysis, the summary RR of colorectal cancer according to high GI remained statistically significant with the exclusion of each study in turn. 

#### 3.2.3. Other Neoplasms

There was no association between high GI or GL intake and lung cancer risk, the summary RRs being 1.11 (95% CI, 0.98–1.26) for GI (six studies, 13,385 cases) and 0.96 (95% CI, 0.87–1.06) for GL (five studies, 12,922 cases) (Figure 3). A significant positive association was found between high GI and bladder cancer, with a summary RR of 1.25 (95% CI, 1.11–1.41; four studies, 3339 cases); the corresponding summary RR for GL was 1.10 (95% CI, 0.85–1.41; four studies, 3339 cases). Summary RRs for kidney cancer were 1.16 (95% CI, 1.02–1.32) for GI (five studies, 4281 cases) and 1.14 (95% CI, 0.80–1.60) for GL (five studies, 4281 cases). Heterogeneity across studies was low-to-moderate in most of the meta-analyses (I^2^ ranging from 0 to 40.5), except for those on GI and lung cancer (I^2^ = 68.2%) and GL and kidney cancer (I^2^ = 78.6%). In sensitivity analyses, the positive association between high GI and bladder cancer remained significant when any study was removed from the meta-analysis, while the summary RR of kidney cancer for high GI was no longer significant with the exclusion of each study in turn, except that by George et al. [53]. 

#### 3.2.4. Subgroup Analyses for Breast Cancer

**Figure 4:**
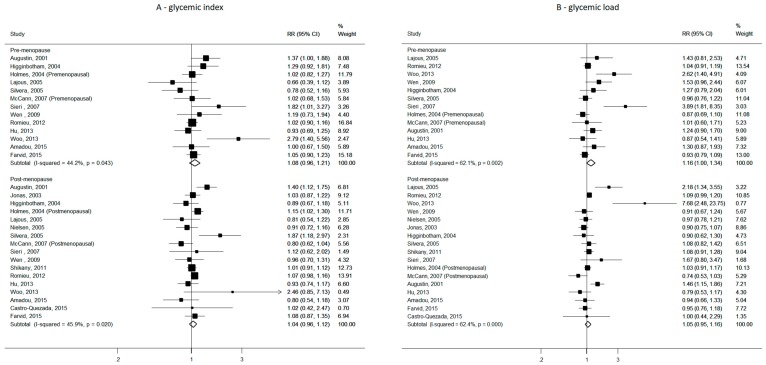
Study-specific and summary relative risks (RRs) of breast cancer for the highest versus the lowest category of glycemic index (**A**) and glycemic load (**B**) in strata of menopausal status.

In subgroup analyses by menopausal status, the summary RRs of breast cancer for the highest versus the lowest category of GI were 1.08 (95% CI, 0.96–1.21, 13 studies, *p* for heterogeneity = 0.043, I^2^= 44.2%) for premenopausal breast cancer and 1.04 (95% CI, 0.96–1.12, 17 studies *p* for heterogeneity = 0.020, I^2^= 45.9%) for postmenopausal breast cancer (*p* for heterogeneity between strata = 0.595) (Figure 4). The corresponding figures for GL were 1.16 (95% CI, 1.00–1.34, 13 studies, *p* for heterogeneity = 0.002, I^2^ = 62.1%) for premenopausal breast cancer and 1.05 (95% CI, 0.95–1.16, 17 studies, *p* for heterogeneity < 0.001, I^2^ = 62.4%) for postmenopausal breast cancer (*p* for heterogeneity between strata = 0.27). 

Summary RRs of breast cancer in strata of BMI indicated a null association with GI and GL among normo-weight as well as overweight women. As for GI, the RRs for the highest versus lowest intake were 1.13 (95% CI, 0.97–1.32, six studies, *p* for heterogeneity = 0.132, I^2^ = 35.8%) for BMI < 25 kg/m^2^ and 0.97 (95% CI, 0.95–1.16, six studies, *p* for heterogeneity = 0.586, I^2^ = 0%) for BMI ≥ 25 kg/m^2^ (*p* for heterogeneity between strata = 0.103). Values for GL were 1.12 (95% CI, 0.94–1.34, seven studies, *p* for heterogeneity = 0.003, I^2^ = 64.3%) for BMI < 25 kg/m^2^ and 0.99 (95% CI, 0.86–1.14, seven studies, *p* for heterogeneity = 0.159, I^2^ = 31.2%) for BMI ≥ 25 kg/m^2^ (*p* for heterogeneity between strata = 0.286).

## 4. Discussion

The present work, based on 88 reports, represents the most updated comprehensive quantification of the relation of GI and GL intake with cancer risk. It updates and expands a previous analysis published in 2015, and provides meta-analytic results for kidney, and for the first time, lung and bladder cancers. The present results largely confirm those of the previous meta-analysis [13]; summary RRs are similar to those previously reported, but, in general, they are more precise, providing more confidence in the results. We found significant, but small, unfavorable effects of high versus low GI diets for colorectal, and possibly bladder and kidney cancers, and a possible modest positive association between high GL and endometrial cancer. Most of the summary RRs were weakly and non-significantly (or at most marginally significantly) increased, indicating no major role of GI/GL intake in the etiology of most cancer types. 

Possible biological mechanisms to explain the modest associations observed include the impact of GI and GL on blood glucose, HbA1c, and hence, insulin and IGF-1. These may also impact on body weight. Although body weight was allowed for in most of the analyses considered, residual confounding may be present. However, GI and GL are correlated with other aspects of carbohydrate quality, such as cereal fibers [54,55]. Confounding by these factors may also partly explain the observed associations.

Our findings are broadly similar to other meta-analyses addressing the role of dietary GI, dietary GL and cancer risk. In particular, no associations with stomach [37], prostate [7,38,39], and pancreatic cancers [56,57,58] were reported by others. A meta-analysis on kidney cancer based on the same study sample yielded essentially identical results [36], i.e., a positive, but weak, significant association with high GI and no association with GL. Although the absence of appreciable heterogeneity among studies supports the robustness of findings on GI and kidney cancer, the still limited number of studies and the null association with GL need to be considered in the interpretation of findings. In addition, the exclusion from the GI-kidney cancer meta-analysis of each study in turn—with the exception of the study by George et al. [53]—gave non-significant summary RR estimates.

According to the World Cancer Research Fund/American Institute for Cancer Research (WCRF/AICR), high dietary GL (but not GI) is probably a cause of endometrial cancer, while evidence on other cancer sites, including colorectal, pancreatic, liver and breast cancers, is inconclusive [59]. The conclusion on endometrial cancer was based on a dose-response meta-analysis based on six cohort studies published up to 2012, which estimated a significant 15% increased risk per 50 units/day of GL. The summary RR of endometrial cancer for high versus low GL was 1.17 (95% CI, 1.00–1.37) in our previous meta-analysis based on 11 studies [13], and 1.12 (95% CI, 0.97–1.56) in the current update, after the inclusion of two additional studies. Although only marginally significant, such 12% increased risk was not in contrast with the WCRF result, thought the different methods of analyses (i.e., dose-response versus extreme quantile), inclusion criteria (e.g., only cohort versus both cohort and case-control studies) and search updates do not allow a direct comparison. A positive association between high GL (but not GI) and the risk of endometrial cancer was reported in other previous meta-analyses [57,60,61], while a recent high profile publication by Reynold et al, which provided systematic reviews and meta-analyses of prospective studies on the relationship between the most widely studied indicators of carbohydrate quality and incidence of, and mortality from, a wide range of non-communicable diseases, did not find any relation with GI and GL, both in dose-response and extreme quantile analyses [7]. In that report, high GI was associated with a marginally significant 10% increased risk of colorectal cancer (summary RR 1.10, 95% CI, 0.99–1.22), and each 10 units/day of GI to a 5% increased risk of borderline significance (summary RR 1.05, 95% CI, 1.00–1.10), while no association was reported for GL [7]. Excess risks of colorectal cancer less than 10% for high versus low GI were found in previous meta-analyses of prospective studies [57,62]. The excess risk was slightly higher (15–20%) —but essentially comparable—in meta-analyses based on both cohort and case-control studies [63,64], including the present one. 

We found a modest nonsignificant association of GI/GL and breast cancer. Other meta-analyses generally showed no or small (<10%) increased risks for the highest versus the lowest GI or GL [7,34,35,57,58,65,66]. In the meta-analyses by Reynolds et al, summary RRs of breast cancer were 1.05 (95% CI, 1.01–1.10) for high versus low GI (but 1.01, 95% CI, 0.98–1.03 for a 10 unit/day increase) and 1.00 (95% CI, 0.95–1.06) for high versus low GL [7]. In another meta-analysis updated to 2015, the association with GI—but not with GL—was slightly stronger in postmenopausal than premenopausal women, but the difference was not significant; in addition, BMI did not influence the association between GI, GL and breast cancer [34]. This is in line with the current results, showing comparable estimates of the GI/GL-breast cancer association in pre- and post-menopausal women, as well as in normo-weight and overweight women. 

No meta-analysis has previously systematically addressed the association of GI/GL with ovarian, lung or bladder cancers. In our study, GI and GL did not significantly influence the risk of lung and ovarian cancer. We found a significant 25% increased risk of bladder cancer for high versus low GI intake, and the association remained significant when excluding each study in turn; no relation was observed for GL. Conditions related to chronic hyperinsulinemia and hyperglycemia, such as diabetes and the metabolic syndrome, have been associated with bladder cancer [10,67]. Urinary tract infections influence the risk of bladder cancer and may be associated with a diet promoting a large increase in blood glucose [68]. In addition, insulin can increase the expression of epidermal growth factors and protein kinase, and induced bladder cancer cell proliferation in in vitro studies [69,70]. 

Part of the different associations of dietary GI and dietary GL on selected cancer types may be explained by differences in the underlying dietary patterns. Some studies indeed suggested that while the overall GL of a diet is mostly associated with high-carbohydrate foods, and hence, closely correlates to total carbohydrate intake, GI is associated not to only to high consumption of high-carbohydrate foods, but also to low consumption of some low-carbohydrate foods (and the related nutrients), including fruit, dairy [71,72], vegetables, and legumes [73]. Thus, dietary GI, unlike GL, may reflect more dimensions of diet than just carbohydrates [74]. This could partly explain at least the positive association of GI with colorectal cancer—a cancer potentially related to various aspects of diet—in the absence of any relation with GL. In any case, in 2015, a scientific consensus statement by international experts on carbohydrate research recognized that the GI is a valid and reproducible measure to express the glycemic response of foods [3].

The heterogeneity in studies’ results observed in most meta-analyses may be due to several reasons, including the dietary habits of the studied populations (i.e., low carbohydrate diets, which include high animal proteins and fats [e.g., for North American populations] versus high carbohydrate diets [e.g., for populations from southern Europe] [75,76,77]), the number and type of carbohydrate items in the FFQs and GI values attributed to FFQ items, with the consequent different ranges of GI/GL (particularly GL) in various studies. Moreover, studies used different methods of GI/GL categorization in the analyses (e.g., tertiles, quartiles, quintiles); therefore, there is heterogeneity in the highest and lowest absolute levels of GI/GL. The study design and the selection of the adjustment factors may also contribute to the observed heterogeneity.

In epidemiologic studies, high dietary GI and GL were consistently associated with greater risks of diabetes and CHD. When restricting our analyses on colorectal and breast cancers to large, well-recognized cohorts which reported on GI/GL and such other health outcomes (i.e., the Swedish Mammography Cohort and cohort of Swedish Men, the Shanghai Women’s and Men’s studies, the Health Professional Follow-up Study, the NHSI and NHSII, the EPIC cohort, the Women’ Health Study and the Women’s Health Initiative), results were essentially unchanged, while heterogeneity decreased for the GI-breast cancer (I^2^ = 0%, *p* = 0.883), GI-colorectal cancer (I^2^ = 9.7%, *p* = 0.353) and GL-breast cancer (I^2^ = 6%, *p* = 0.384) meta-analyses, but not appreciably for the meta-analysis on GL and colorectal cancer (I^2^ = 71.2%, *p* = 0.001). 

Most studies adjusted for the most relevant confounders, i.e., BMI/physical activity, energy intake, tobacco and social class, but some residual confounding is still possible. GI/GL are positively associated with diabetes, and diabetes increases the risk of colorectal, pancreatic, endometrial and perhaps breast cancer [78] (and possibly decreases the risk of prostate cancer) [79]. In addition, diabetic patients are often advised to change their diet to lower carbohydrate intake, and this could underestimate any positive association. Therefore, careful consideration of subjects’ diabetes condition is necessary when examining the relation of GI and GL with diabetes-related neoplasms. In most studies on colorectal, endometrial and prostate cancer, authors either: (1) excluded diabetics from the analyses; (2) gave RRs adjusted for diabetes; (3) reported that adjustment for diabetes or restricting the analyses to non-diabetic subjects did not influence the results; or (4) reported that the observed association was independent from diabetes. However, excluding women with diabetes slightly strengthened the association between GI and colorectal cancer risk in the Swedish Mammography Cohort [80], and higher GL was positively associated with endometrial cancer among nondiabetic women only in the Iowa Women’s Health Study [81].

Major strengths of the present study are the consideration of many studies and cancer cases, which provided statistical power to detect moderate associations for most cancer sites, the use of a rigorous and systematic methodology for identifying and pooling evidence from previous studies, and the ability to examine GI and GL in association with several types of cancer from cohort and case-control studies in a single report.

## 5. Conclusions

The present analysis provides the most comprehensive and updated quantification of the relation between GI, GL and cancer risk. It indicates moderate unfavorable effects of high versus low GI on colorectal, and possibly, bladder and kidney cancers, and a possible modest positive association between GL and endometrial cancer. Such small excess risks may, however, be relevant at the population level, due to the high incidence of selected cancers, namely colorectal and breast cancer. 

## Figures and Tables

**Table 1:** Main characteristics of the studies included in the systematic review.

Study^, Year (Sex)	Cancer Site	Study Design Study Area and Period of Enrolment or Follow-Up (Years)	Cases	Controls/Cohort size	Dietary Assessment Method; Reference Food for GI/GL	RR (95% CI) Comparison Level (Highest vs. Lowest)		Matching and Adjustment Factors
						Glycemic Index	Glycemic Load	
Li 2017 [24] (M + W) *US Multicenter Study (1)* *L.A. Multi-Ethnic study (2)*	EA, GCA	CC, pb USA, in (1): 1993-1995 in (2): 1992-1997	500 EA 529 GCA	2027	in (1): Validated 104-items FFQ in (2): validated 124-items FFQ; not specified	EA OR: 1.58 (1.13–2.21) V vs. I study-specific quintile GCA OR: 1.21 (0.88–1.67) V vs. I study-specific quintile	EA OR: 0.81 (0.51–1.29) V vs. I study-specific quintile GCA OR: 0.86 (0.55–1.35) V vs. I study-specific quintile	Age, sex, study, fruit and vegetables, smoking, GERD, energy, BMI (only for GL)
Haluszka 2019 [30] (M + W)	CRC	CC, pb Argentina 2008-2016	161	331	validated 127-items FFQ; white bread	OR: 1.08 (0.61–1.90) > 82.5 vs. <77.2 III vs. I tertile	OR: 1.64 (1.16–2.34) >298.7 vs. <200.4 III vs. I tertile	Age, sex, socio-economic status, urbanization, BMI, smoking, analgesic use, family history,
Huang 2018 [31] (M + W)	CRC	CC, hb and pb China, 2010-2017	1944	2027 (1168 hb, 859 pb)	validated 81-items FFQ; glucose	OR: 3.10 (2.51–3.85) >69.8 vs. <64.3 IV vs. I quartile	OR: 1.14 (0.94–1.39) >187.2 vs. <134.4 IV vs. I quartile	Age, sex, marital status, residence, education, occupation, income, smoking, passive smoking, alcohol, family history of cancer, occupational physical activity, household and leisure-time activities, energy
Abe 2016 [17] *JPHC Study* (M, W)	CRC	Cohort Japan, 1995/1999–2010 average FU: 12.5 years	1468	73,501 pr 919,276 py	validated 128-items FFQ; not specified	RR, M: 0.92 (9.73–1.14) 65.77-78.46 vs. 28.12-59.10 IV vs. I quartile RR, W: 0.97 (0.73–1.30) 64.05-80.19 vs. 14.25-57.57 IV vs. I quartile	RR, M: 0.79 (0.58–1.08) IV vs. I quartile RR, W: 0.82 (0.55–1.24) IV vs. I quartile	Stratified by sex and adjusted for age, area, alcohol, smoking, BMI, MET, history of diabetes, colorectal screening, calcium, magnesium, vitamin B6, vitamin B12, folate, vitamin D, n-3 PUFA, fiber, red meat, and for women only, menopausal status and use of exogenous female hormones.
Larsson 2016 [19] *SMC* and *CSM* (M+W)	Biliary tract	Cohort Sweden, 1998–2012 mean FU: 13.3 years	163 ^#^	76,014 pr 1,010,777 py	validated 96-items FFQ; white bread	Total extrahepatic BTC (*n* = 140): RR: 2.12 (1.25–3.58) Gallbladder (*n* = 77): RR: 1.58 (0.81–3.08) Intrahepatic BTC (*n* = 23) RR: 1.47 (0.54–3.97) IV (median: 88 in M and 80 in W) vs. I (median: 73 in M and 69 in W) quartile	Total extrahepatic BTC (*n* = 140): RR: 1.63 (1.01–2.63) Gallbladder (*n* = 77): RR: 2.14 (1.06–4.33) Intrahepatic BTC (*n* = 23) RR: 3.46 (1.22–9.84) M: >213 vs. <177 W: >202 vs. <166 IV vs. I (sex-specific) quartile	Age, sex, education, smoking, BMI, diabetes, energy
Sun 2018 [25] (M, W) *SWHS* and *SMHS*	Lung	Cohort Shanghai, 1997/2000–2013 in SWHS 2002/2006–2013 in SMHS average FU: 14.8 years in SWHS and 9.3 years in SMHS	1312 (649 in SWHS and 663 in SMHS)	130,852 pr 1.612,703 py	SWHS: validated 77-items FFQ SMHS: validated 81-items FFQ; not reported	RR, W (SWHS): 1.16 (0.92–1.47) IV (median 76.74) vs. I (median 63.63) quartile RR, M (SMHS): 0.83 (0.67–1.03) IV (median 77.02) vs. I (median 64.13) quartile	RR, W (SWHS): 1.09 (0.86–1.37) IV (median 144.8 g/1000 kcal/d) vs. I (median 97.72 g/1000 kcal/d) quartile RR, M (SMHS): 0.85 (0.68–1.05) IV (median 143.77 g/1000 kcal/d) vs. I (median 95.79 g/1000 kcal/d) quartile	Age, education, income, BMI, physical activity, energy, smoking, alcohol (men only), history of lung disease, hypertension, diabetes, family history of cancer, menopausal status (women only)
Melkonian 2016 [22] (M+W)	Lung	CC, hb USA, not reported	1905	2413	validated FFQ; glucose	OR: 1.49 (1.21–1.83) V vs. I (sex-specific) quintile	OR: 1.16 (0.94–1.42) V vs. I (sex-specific) quintile	Age, education, gender, smoking, history of emphysema, pneumonia, hay fever, family history of lung cancer, physical activity, BMI, energy
Malavolti 2017 [23] (M+W)	Melanoma	CC, pb Italy, 2005–2006	380	719	validated 188-items FFQ; glucose	OR: 0.88 (0.55–1.42) V (median: 55.7) vs. I (median: 47.7) quintile	OR: 1.35 (0.80–2.27) V (median:149.7) vs. I (median: 92.0) quintile	Age, sex, residence, education, BMI, phototype, skin sensitivity to sun exposure, sunburns history, SFA, vitamin C, vitamin D, fiber, energy
Guerrero 2019 [32] (W)	Breast	CC, pb Mexico, 2007–2011	509	509	validated 133-items FFQ; glucose	OR: 1.15 (1.09–1.23) each unit increment	OR: 1.00 (0.99–1.02) each unit increment	Age, education, energy, saturated fats, breastfeeding duration
Castro-Quezada 2016 [14] *PREDIMED* * (W)	Breast	Cohort Spain, 2003/2009–2010 median FU: 4.8 years	32	4010 pr (postmenopausal W) 17,757 py	validated 137-items FFQ; glucose	RR: 1.02 (0.42–2.46) III (mean: 60.8) vs. I (mean: 50.8) tertile	RR: 1.00 (0.44–2.30) III (mean: 129.0) vs. I (mean: 82.2) tertile	Age, center, intervention group, smoking, education, physical activity, BMI, WHtR, family history of cancer, age at menopause, HRT use, statin use, energy, alcohol, dietary fiber, folate
Amadou 2015 [15] *Cáncer de la Mamá study* (W)	Breast	CC, pb Mexico, 2004–2007	1000	1074	validated 104-items FFQ; glucose	OR: 0.90 (0.68–1.12) >52.5 vs. <46.8 IV vs. I quartile Premenopausal W: 1.0 (0.67–1.5) Postmenopausal W: 0.80 (0.55–1.2)	OR: 1.1 (0.82–1.1) >173 vs. <145.8 IV vs. I quartile Premenopausal W: 1.3 (0.86–1.9) Postmenopausal W: 0.94 (0.65–1.3)	Age, age at menarche, SES, breastfeeding, age at first pregnancy, family history of breast cancer, alcohol, physical activity, energy, native ancestry, BMI
Farvid 2015 [16] *NHSII* (W)	Breast	Cohort USA, 1991–2011	2833	90,534 pr 1,725,295 py	validated 128-items FFQ; glucose	RR: 1.03 (0.91–1.16) V (median: 57.9) vs. I (median: 49.7) quintile Premenopausal W RR: 1.05 (0.90–1.23) Postmenopausal W RR: 1.08 (0.87–1.35)	RR: 0.94 (0.83–1.06) V (median: 149) vs. I (median: 96) quintile Premenopausal W RR: 0.93 (0.79–1.09) Postmenopausal W RR: 0.95 (0.76–1.18)	Age, race, family history of breast cancer, history of BBD, smoking, height, BMI at 18 years, weight change since age 18 years, age at menarche, parity and age at first birth, OC use, alcohol, energy, menopausal status and age at menopause, postmenopausal hormone use.
Hartman 2018 [33] *CPS-II Nutrition Cohort* (W)	Endometrium	Cohort USA, 1999–2013 average FU: 13.6 years	425	30,997 pr postmenopausal W 377,265 py	validated 152-items FFQ; not specified	RR: 0.98 (0.74–1.29) ≥54.48 vs. <50.43 IV vs. I quartile	RR: 0.83 (0.62–1.11) ≥126.82 vs. <100.47 IV vs. I quartile	Age, smoking, age at menarche, age at menopause, parity, HRT, OC, physical activity, BMI
Brenner 2015 [20] (W)	Endometrium	CC, pb Canada, 2002-2006	511	980	adapted NCI DHQ (validated); not specified	Not provided	OR: 0.87 (0.52–1.46) >114.1 vs. ≤68.7 IV vs. I quartile	Age, parity, menopausal status, HRT, rural residence, weight, waist circumference, comorbidity (diabetes, hypertension, thrombosis, pulmonary embolism, myocardial infarction, angina pectoris, stroke, high cholesterol), fiber, energy
Qin 2016 [21] *AACES* (W)	Ovary	CC, pb USA, 2010–2014	406	609	Block 2005 FFQ (110 items, validated); glucose	OR: 1.03 (0.70–1.50) ≥54.9 vs. ≤49.9 IV vs. I quartile	OR: 1.35 (0.93–1.97) ≥65.0 vs. ≤50.8 units/1000 kcal IV vs. I quartile	Age, education, region, energy, parity, OC use, menopausal status, tubal ligation, family history of breast/ovarian cancer
Augustin 2017 [28] (M+W)	Bladder	CC, hb Italy, 2003–2014	578	608	validated 78-items FFQ; white bread	OR: 1.18 (0.83–1.67) >83 vs. <75 IV vs. I quartile	OR: 1.96 (1.16–3.31) ≥275 vs. <169 IV vs. I quartile	Age, sex, study center, education, smoking, alcohol, abdominal obesity, energy
Zhu 2017 [27] (M+W)	Kidney	CC, pb USA, 2002–2017 (ongoing)	854	1255	validated FFQ; glucose	OR: 1.32 (0.99–1.74) IV vs. I (sex-specific) quartile	OR: 1.15 (0.88–1.51) IV vs. I (sex-specific) quartile	Age, sex, education, BMI, physical activity, smoking, hypertension, family history, energy, HEI-2015
Zamora-Ros 2016 [18] *EPIC* (M+W)	Thyroid	Cohort 10 European countries, 1992/2000–2006/2009 mean FU: 11 years	556	477,274 pr 5,262,772 py	country-specific validated dietary questionnaires;glucose	RR: 0.94 (0.73–1.20) >58.5 vs. <53.6 IV vs. I (cohort-wide) quartile	RR: 0.95 (0.74–1.24) >69.6 vs. <54.4 unit/1000 kcal day IV vs. I (cohort-wide) quartile	Age, sex, center, BMI, smoking, education, physical activity, energy, alcohol, and for women only, menopausal status and type
Makarem 2017 [29] (M+W) *Framingham Offspring cohort*	Adiposity-related cancers	Cohort USA, 1991/1995-2013	565 adiposity-related cancers^§^ 124 breast 157 prostate 68 CRC	3184 pr	validated 126-items FFQ; not specified	Adiposity-related cancers RR: 0.95 (0.73–1.24) >57.5 vs. <51.9 Breast RR: 0.90 (0.59–.37) >56.2 vs. <53.3 III vs. I tertile Prostate RR: 0.74 (0.48–1.12) >56.4 vs. <53.6 III vs. I tertile CRC RR: 1.51 (0.81–2.84) >56.3 vs. <53.5 III vs. I tertile	Adiposity-related cancers RR: 0.93 (0.58–1.49) >169.9 vs. <85.6 Breast RR: 0.54 (0.26–1.09) >136.0 vs. <96.7 g/d III vs. I tertile Prostate RR: 0.76 (0.40–1.43) >154.4 vs. <106.3 g/d III vs. I tertile CRC RR: 1.21 (0.43–3.40) >143.7 vs. <100.7 g/d III vs. I tertile	Age, sex, smoking, alcohol, energy Breast: additional adjustment for menopausal status, age at menopause, hormone therapy, n. of live births. CRC: additional adjustment for red and processed meat, fiber For breast, prostate and CRC: additional adjustment for education, BMI, waist circumference, physical activity, history of diabetes and CVD, and antioxidant supplements did not change the results.
Sieri 2017 [26] (M+W) *EPIC-Italy*	Various sites	Cohort Italy 1993/1998–2009/2010 median FU: 14.9 years	5112 cancers	45,148 pr	3 validated FFQs; glucose	All cancers combined RR: 1.06 (0.97–1.16) V (mean: 57.4) vs. I (mean: 50.0) quintile The paper reported RRs of 20 different cancer sites	All cancers combined RR: 1.05 (0.93–1.20) V (mean: 235.2) vs. I (mean: 86.0) quintile The paper reported RRs of 20 different cancer sites	Age, sex, education, BMI, physical activity, smoking, FFQ, alcohol, non-alcohol energy, fiber, saturated fat

Abbreviations: AACES, African American Cancer Epidemiology Study; BBD, benign breast disease; BMI, body mass index; BTC, biliary tract cancer; CC, case-control; CI, confidence interval; CPS, Cancer Prevention Study; CRC, colorectum; CSM, cohort of Swedish Men; CVD, cardiovascular disease; DHQ, Diet History Questionnaire; EA: Esophageal adenocarcinoma; EPIC, European Prospective Investigation into Cancer and nutrition; FFQ, food frequency questionnaire; FU, follow-up; GCA, gastric cardia adenocarcinoma; GERD, gastro-esophageal reflux disease; GI, glycemic index; GL, glycemic load; hb, hospital-based; HEI, Healthy Eating Index; JPHC, Japan Public Health Center-based; HRT, hormone replacement therapy; M, men; MET, metabolic equivalent tasks; NCI, National Cancer Institute; OC, oral contraceptives; OR, odds ratio; pb, population-based; pr, persons at risk; PREDIMED, PREvención con DIeta MEDiterránea; py, person-years; PUFA, polyunsaturated fatty acids; RR, relative risk; SFA, saturated fatty acids; SMC, Swedish Mammography Cohort; SMHS, Shanghai Men’s Health Study; SWHS, Shanghai Women’s Health Study; W, women; WHtR, waist-to-height ratio.^ Study’s name is indicated in Italic. # 140 extrahepatic (including 77 gallbladder cancers) and 23 intrahepatic biliary tract cancers. * Observational cohort of postmenopausal women at high risk of cardiovascular diseases participating in a randomized, parallel group, clinical trial (i.e., PREDIMED trial). § adiposity-related cancers included cancers of the gastrointestinal tract, reticuloendothelial system (blood, bone and spleen), female reproductive tracts, genitourinary organs and the thyroid gland.

## Supplementary Materials

The following are available online at https://www.mdpi.com/2072-6643/11/10/2342/s1, Supplementary Material: References of studies included in the meta-analyses by cancer site (in alphabetical order).
