# Supplementary material for: Glycemic Index, Glycemic Load and Cancer Risk: An Updated Meta-Analysis
Source: Nutrients. 2019 Oct 2;11(10):2342. doi: 10.3390/nu11102342 (PMC6835610; doi:10.3390/nu11102342)
Supplement: Supplementary file 1 [file nutrients-11-02342-s001.pdf]

Supplementary Material

Manuscript: "Glycemic index, glycemic load and cancer risk: an updated meta-analysis"

References of studies included in the meta-analyses by cancer site (in alphabetical order)

1. Hormone-related cancers

1.1 Breast cancer

Amadou A, Degoul J, Hainaut P, Chajes V, Biessy C, Torres Mejia G, Huybrechts I, Moreno Macia H, Ortega C, Angeles-Llerenas A, Romieu I (2015) Dietary Carbohydrate, Glycemic Index, Glycemic Load, and Breast Cancer Risk Among Mexican Women. *Epidemiology* **26**(6)**:** 917-24

Augustin LS, Dal Maso L, La Vecchia C, Parpinel M, Negri E, Vaccarella S, Kendall CW, Jenkins DJ, Franceschi S (2001) Dietary glycemic index and glycemic load, and breast cancer risk: a case-control study. *Ann Oncol* **12**(11)**:** 1533-8

Castro-Quezada I, Sanchez-Villegas A, Martinez-Gonzalez MA, Salas-Salvado J, Corella D, Estruch R, Schroder H, Alvarez-Perez J, Ruiz-Lopez MD, Artacho R, Ros E, Bullo M, Sorli JV, Fito M, Ruiz-Gutierrez V, Toledo E, Buil-Cosiales P, Garcia Rodriguez A, Lapetra J, Pinto X, Salaverria I, Tur JA, Romaguera D, Tresserra-Rimbau A, Serra-Majem L, Investigators PS (2016) Glycemic index, glycemic load and invasive breast cancer incidence in postmenopausal women: The PREDIMED study. *Eur J Cancer Prev* **25**(6)**:** 524-32

Farvid MS, Eliassen AH, Cho E, Chen WY, Willett WC (2015) Adolescent and Early Adulthood Dietary Carbohydrate Quantity and Quality in Relation to Breast Cancer Risk. *Cancer Epidemiol Biomarkers Prev* **24**(7)**:** 1111-20

George SM, Mayne ST, Leitzmann MF, Park Y, Schatzkin A, Flood A, Hollenbeck A, Subar AF (2009) Dietary glycemic index, glycemic load, and risk of cancer: a prospective cohort study. *Am J Epidemiol* **169**(4)**:** 462-72

Higginbotham S, Zhang ZF, Lee IM, Cook NR, Buring JE, Liu S (2004) Dietary glycemic load and breast cancer risk in the Women's Health Study. *Cancer Epidemiol Biomarkers Prev* **13**(1)**:** 65-70

Holmes MD, Liu S, Hankinson SE, Colditz GA, Hunter DJ, Willett WC (2004) Dietary carbohydrates, fiber, and breast cancer risk. *Am J Epidemiol* **159**(8)**:** 732-9

Hu J, La Vecchia C, Augustin LS, Negri E, de Groh M, Morrison H, Mery L (2013) Glycemic index, glycemic load and cancer risk. *Ann Oncol* **24**(1)**:** 245-51

Jonas CR, McCullough ML, Teras LR, Walker-Thurmond KA, Thun MJ, Calle EE (2003) Dietary glycemic index, glycemic load, and risk of incident breast cancer in postmenopausal women. *Cancer Epidemiol Biomarkers Prev* **12**(6)**:** 573-7

Lajous M, Willett W, Lazcano-Ponce E, Sanchez-Zamorano LM, Hernandez-Avila M, Romieu I (2005) Glycemic load, glycemic index, and the risk of breast cancer among Mexican women. *Cancer Causes Control* **16**(10)**:** 1165-9

Larsson SC, Bergkvist L, Wolk A (2009) Glycemic load, glycemic index and breast cancer risk in a prospective cohort of Swedish women. *Int J Cancer* **125**(1)**:** 153-7

Levi F, Pasche C, Lucchini F, Bosetti C, La Vecchia C (2002) Glycaemic index, breast and colorectal cancer. *Ann Oncol* **13**(10)**:** 1688-9

Makarem N, Bandera EV, Lin Y, Jacques PF, Hayes RB, Parekh N (2017) Carbohydrate nutrition and risk of adiposity-related cancers: results from the Framingham Offspring cohort (1991-2013). *Br J Nutr* **117**(11)**:** 1603-1614

McCann SE, McCann WE, Hong CC, Marshall JR, Edge SB, Trevisan M, Muti P, Freudenheim JL (2007) Dietary patterns related to glycemic index and load and risk of premenopausal and postmenopausal breast cancer in the Western New York Exposure and Breast Cancer Study. *Am J Clin Nutr* **86**(2)**:** 465-71

Nielsen TG, Olsen A, Christensen J, Overvad K, Tjonneland A (2005) Dietary carbohydrate intake is not associated with the breast cancer incidence rate ratio in postmenopausal Danish women. *J Nutr* **135**(1)**:** 124-8

Romieu I, Ferrari P, Rinaldi S, Slimani N, Jenab M, Olsen A, Tjonneland A, Overvad K, Boutron-Ruault MC, Lajous M, Kaaks R, Teucher B, Boeing H, Trichopoulou A, Naska A, Vasilopoulo E, Sacerdote C, Tumino R, Masala G, Sieri S, Panico S, Bueno-de-Mesquita HB, Van-der AD, van Gils CH, Peeters PH, Lund E, Skeie G, Asli LA, Rodriguez L, Navarro C, Amiano P, Sanchez MJ, Barricarte A, Buckland G, Sonestedt E, Wirfalt E, Hallmans G, Johansson I, Key TJ, Allen NE, Khaw KT, Wareham NJ, Norat T, Riboli E, Clavel-Chapelon F (2012) Dietary glycemic index and glycemic load and breast cancer risk in the European Prospective Investigation into Cancer and Nutrition (EPIC). *Am J Clin Nutr* **96**(2)**:** 345-55

Shikany JM, Redden DT, Neuhouser ML, Chlebowski RT, Rohan TE, Simon MS, Liu S, Lane DS, Tinker L (2011) Dietary glycemic load, glycemic index, and carbohydrate and risk of breast cancer in the Women's Health Initiative. *Nutr Cancer* **63**(6)**:** 899-907

Sieri S, Pala V, Brighenti F, Pellegrini N, Muti P, Micheli A, Evangelista A, Grioni S, Contiero P, Berrino F, Krogh V (2007) Dietary glycemic index, glycemic load, and the risk of breast cancer in an Italian prospective cohort study. *Am J Clin Nutr* **86**(4)**:** 1160-6

Silvera SA, Jain M, Howe GR, Miller AB, Rohan TE (2005) Dietary carbohydrates and breast cancer risk: a prospective study of the roles of overall glycemic index and glycemic load. *Int J Cancer* **114**(4)**:** 653-8

Wen W, Shu XO, Li H, Yang G, Ji BT, Cai H, Gao YT, Zheng W (2009) Dietary carbohydrates, fiber, and breast cancer risk in Chinese women. *Am J Clin Nutr* **89**(1)**:** 283-9

Woo HD, Park KS, Shin A, Ro J, Kim J (2013) Glycemic Index and Glycemic Load Dietary Patterns and the Associated Risk of Breast Cancer: A Case-control Study. *Asian Pac J Cancer Prev* **14**(9)**:** 5193-8

Yun SH, Kim K, Nam SJ, Kong G, Kim MK (2010) The association of carbohydrate intake, glycemic load, glycemic index, and selected rice foods with breast cancer risk: a case-control study in South Korea. *Asia Pac J Clin Nutr* **19**(3)**:** 383-92

1.2 Endometrium

Augustin LS, Gallus S, Bosetti C, Levi F, Negri E, Franceschi S, Dal Maso L, Jenkins DJ, Kendall CW, La Vecchia C (2003) Glycemic index and glycemic load in endometrial cancer. *Int J Cancer* **105**(3)**:** 404-7

Brenner DR, Speidel T, Csizmadi I, Biel RK, Cook LS, Courneya KS, Friedenreich CM (2015) Glycemic load and endometrial cancer risk in a case-control study of Canadian women. *Cancer Epidemiol* **39**(2)**:** 170-3

Coleman HG, Kitahara CM, Murray LJ, Dodd KW, Black A, Stolzenberg-Solomon RZ, Cantwell MM (2014) Dietary Carbohydrate Intake, Glycemic Index, and Glycemic Load and Endometrial Cancer Risk: A Prospective Cohort Study. *Am J Epidemiol* **179**(1)**:** 75-84

Cust AE, Slimani N, Kaaks R, van Bakel M, Biessy C, Ferrari P, Laville M, Tjonneland A, Olsen A, Overvad K, Lajous M, Clavel-Chapelon F, Boutron-Ruault MC, Linseisen J, Rohrmann S, Nothlings U, Boeing H, Palli D, Sieri S, Panico S, Tumino R, Sacerdote C, Skeie G, Engeset D, Gram IT, Quiros JR, Jakszyn P, Sanchez MJ, Larranaga N, Navarro C, Ardanaz E, Wirfalt E, Berglund G, Lundin E, Hallmans G, Bueno-de-Mesquita HB, Du H, Peeters PH, Bingham S, Khaw KT, Allen NE, Key TJ, Jenab M, Riboli E (2007) Dietary carbohydrates, glycemic index, glycemic load, and endometrial cancer risk within the European Prospective Investigation into Cancer and Nutrition cohort. *Am J Epidemiol* **166**(8)**:** 912-23

Folsom AR, Demissie Z, Harnack L (2003) Glycemic index, glycemic load, and incidence of endometrial cancer: the Iowa women's health study. *Nutr Cancer* **46**(2)**:** 119-24

Galeone C, Augustin LS, Filomeno M, Malerba S, Zucchetto A, Pelucchi C, Montella M, Talamini R, Franceschi S, La Vecchia C (2013) Dietary glycemic index, glycemic load, and the risk of endometrial cancer: a case-control study and meta-analysis. *Eur J Cancer Prev* **22**(1)**:** 38-45

George SM, Mayne ST, Leitzmann MF, Park Y, Schatzkin A, Flood A, Hollenbeck A, Subar AF (2009) Dietary glycemic index, glycemic load, and risk of cancer: a prospective cohort study. *Am J Epidemiol* **169**(4)**:** 462-72

Hartman TJ, McCullough ML, Hodge JM, Gaudet MM, Wang Y, Gapstur SM (2018) Dietary Energy Density, Glycemic Load, Glycemic Index, and Risk for Endometrial Cancer in the CPS-II Nutrition Cohort. *Cancer Epidemiol Biomarkers Prev* **27**(1)**:** 113-115

Larsson SC, Friberg E, Wolk A (2007) Carbohydrate intake, glycemic index and glycemic load in relation to risk of endometrial cancer: A prospective study of Swedish women. *Int J Cancer* **120**(5)**:** 1103-7

Nagle CM, Olsen CM, Ibiebele TI, Spurdle AB, Webb PM (2013) Glycemic index, glycemic load and endometrial cancer risk: results from the Australian National Endometrial Cancer study and an updated systematic review and meta-analysis. *Eur J Nutr* **52**(2)**:** 705-15

Silvera SA, Rohan TE, Jain M, Terry PD, Howe GR, Miller AB (2005) Glycaemic index, glycaemic load and risk of endometrial cancer: a prospective cohort study. *Public Health Nutr* **8**(7)**:** 912-9

Xu WH, Xiang YB, Zhang X, Ruan Z, Cai H, Zheng W, Shu XO (2015) Association of dietary glycemic index and glycemic load with endometrial cancer risk among chinese women. *Nutr Cancer* **67**(1)**:** 89-97

1.3 Ovary

Augustin LS, Polesel J, Bosetti C, Kendall CW, La Vecchia C, Parpinel M, Conti E, Montella M, Franceschi S, Jenkins DJ, Dal Maso L (2003) Dietary glycemic index, glycemic load and ovarian cancer risk: a case-control study in Italy. Ann Oncol 14(1): 78-84

George SM, Mayne ST, Leitzmann MF, Park Y, Schatzkin A, Flood A, Hollenbeck A, Subar AF (2009) Dietary glycemic index, glycemic load, and risk of cancer: a prospective cohort study. Am J Epidemiol 169(4): 462-72

Hu J, La Vecchia C, Augustin LS, Negri E, de Groh M, Morrison H, Mery L (2013) Glycemic index, glycemic load and cancer risk. Ann Oncol 24(1): 245-51

King MG, Olson SH, Paddock L, Chandran U, Demissie K, Lu SE, Parekh N, Rodriguez-Rodriguez L, Bandera EV (2013) Sugary food and beverage consumption and epithelial ovarian cancer risk: a population-based case-control study. BMC Cancer 13: 94

Nagle CM, Kolahdooz F, Ibiebele TI, Olsen CM, Lahmann PH, Green AC, Webb PM (2011) Carbohydrate intake, glycemic load, glycemic index, and risk of ovarian cancer. Ann Oncol 22(6): 1332-8

Qin B, Moorman PG, Alberg AJ, Barnholtz-Sloan JS, Bondy M, Cote ML, Funkhouser E, Peters ES, Schwartz AG, Terry P, Schildkraut JM, Bandera EV (2016) Dietary carbohydrate intake, glycaemic load, glycaemic index and ovarian cancer risk in African-American women. Br J Nutr 115(4): 694-702

Sieri S, Agnoli C, Pala V, Grioni S, Brighenti F, Pellegrini N, Masala G, Palli D, Mattiello A, Panico S, Ricceri F, Fasanelli F, Frasca G, Tumino R, Krogh V (2017) Dietary glycemic index, glycemic load, and cancer risk: results from the EPIC-Italy study. Sci Rep 7(1): 9757

Silvera SA, Jain M, Howe GR, Miller AB, Rohan TE (2007) Glycaemic index, glycaemic load and ovarian cancer risk: a prospective cohort study. Public Health Nutr 10(10): 1076-81

1.4 Prostate

Augustin LS, Galeone C, Dal Maso L, Pelucchi C, Ramazzotti V, Jenkins DJ, Montella M, Talamini R, Negri E, Franceschi S, La Vecchia C (2004) Glycemic index, glycemic load and risk of prostate cancer. Int J Cancer 112(3): 446-50

George SM, Mayne ST, Leitzmann MF, Park Y, Schatzkin A, Flood A, Hollenbeck A, Subar AF (2009) Dietary glycemic index, glycemic load, and risk of cancer: a prospective cohort study. Am J Epidemiol 169(4): 462-72

Hu J, La Vecchia C, Augustin LS, Negri E, de Groh M, Morrison H, Mery L (2013) Glycemic index, glycemic load and cancer risk. Ann Oncol 24(1): 245-51

Makarem N, Bandera EV, Lin Y, Jacques PF, Hayes RB, Parekh N (2017) Carbohydrate nutrition and risk of adiposity-related cancers: results from the Framingham Offspring cohort (1991-2013). Br J Nutr 117(11): 1603-1614

Nimptsch K, Kenfield S, Jensen MK, Stampfer MJ, Franz M, Sampson L, Brand-Miller JC, Willett WC, Giovannucci E (2011) Dietary glycemic index, glycemic load, insulin index, fiber and whole-grain intake in relation to risk of prostate cancer. Cancer Causes Control 22(1): 51-61

Shikany JM, Flood AP, Kitahara CM, Hsing AW, Meyer TE, Willcox BJ, Redden DT, Ziegler RG (2011) Dietary carbohydrate, glycemic index, glycemic load, and risk of prostate cancer in the Prostate, Lung, Colorectal, and Ovarian Cancer Screening Trial (PLCO) cohort. Cancer Causes Control 22(7): 995-1002

Sieri S, Agnoli C, Pala V, Grioni S, Brighenti F, Pellegrini N, Masala G, Palli D, Mattiello A, Panico S, Ricceri F, Fasanelli F, Frasca G, Tumino R, Krogh V (2017) Dietary glycemic index, glycemic load, and cancer risk: results from the EPIC-Italy study. Sci Rep 7(1): 9757

Vidal AC, Williams CD, Allott EH, Howard LE, Grant DJ, McPhail M, Sourbeer KN, Hwa LP, Boffetta P, Hoyo C, Freedland SJ (2015) Carbohydrate intake, glycemic index and prostate cancer risk. Prostate 75(4): 430-9

2. Digestive-tract cancers

2.1 Stomach

Augustin LS, Gallus S, Negri E, La Vecchia C (2004) Glycemic index, glycemic load and risk of gastric cancer. Ann Oncol 15(4): 581-4

Bertuccio P, Praud D, Chatenoud L, Lucenteforte E, Bosetti C, Pelucchi C, Rossi M, Negri E, La Vecchia C (2009) Dietary glycemic load and gastric cancer risk in Italy. Br J Cancer 100(3): 558-61

George SM, Mayne ST, Leitzmann MF, Park Y, Schatzkin A, Flood A, Hollenbeck A, Subar AF (2009) Dietary glycemic index, glycemic load, and risk of cancer: a prospective cohort study. Am J Epidemiol 169(4): 462-72

Hu J, La Vecchia C, Augustin LS, Negri E, de Groh M, Morrison H, Mery L (2013) Glycemic index, glycemic load and cancer risk. Ann Oncol 24(1): 245-51

Larsson SC, Bergkvist L, Wolk A (2006) Glycemic load, glycemic index and carbohydrate intake in relation to risk of stomach cancer: a prospective study. Int J Cancer 118(12): 3167-9

Lazarevic K, Nagorni A, Jeremic M (2009) Carbohydrate intake, glycemic index, glycemic load and risk of gastric cancer. Cent Eur J Public Health 17(2): 75-8

2.2 Colorectum

Abe SK, Inoue M, Sawada N, Ishihara J, Iwasaki M, Yamaji T, Shimazu T, Sasazuki S, Tsugane S (2016) Glycemic index and glycemic load and risk of colorectal cancer: a population-based cohort study (JPHC Study). Cancer Causes Control 27(4): 583-93

Franceschi S, Dal Maso L, Augustin L, Negri E, Parpinel M, Boyle P, Jenkins DJ, La Vecchia C (2001) Dietary glycemic load and colorectal cancer risk. Ann Oncol 12(2): 173-8

George SM, Mayne ST, Leitzmann MF, Park Y, Schatzkin A, Flood A, Hollenbeck A, Subar AF (2009) Dietary glycemic index, glycemic load, and risk of cancer: a prospective cohort study. Am J Epidemiol 169(4): 462-72

Haluszka E, Davila VL, Aballay LR, Del Pilar Diaz M, Osella AR, Niclis C (2019) Association of the glycaemic index and the glycaemic load with colorectal cancer in the population of Cordoba (Argentina): results of a case-control study using a multilevel modelling approach. Br J Nutr: 1-22

Higginbotham S, Zhang ZF, Lee IM, Cook NR, Giovannucci E, Buring JE, Liu S (2004) Dietary glycemic load and risk of colorectal cancer in the Women's Health Study. J Natl Cancer Inst 96(3): 229-33

Howarth NC, Murphy SP, Wilkens LR, Henderson BE, Kolonel LN (2008) The association of glycemic load and carbohydrate intake with colorectal cancer risk in the Multiethnic Cohort Study. Am J Clin Nutr 88(4): 1074-82

Hu J, La Vecchia C, Augustin LS, Negri E, de Groh M, Morrison H, Mery L (2013) Glycemic index, glycemic load and cancer risk. Ann Oncol 24(1): 245-51

Huang J, Fang YJ, Xu M, Luo H, Zhang NQ, Huang WQ, Pan ZZ, Chen YM, Zhang CX (2018) Carbohydrate, dietary glycaemic index and glycaemic load, and colorectal cancer risk: a case-control study in China. Br J Nutr 119(8): 937-948

Kabat GC, Shikany JM, Beresford SA, Caan B, Neuhouser ML, Tinker LF, Rohan TE (2008) Dietary carbohydrate, glycemic index, and glycemic load in relation to colorectal cancer risk in the Women's Health Initiative. Cancer Causes Control 19(10): 1291-8

Larsson SC, Giovannucci E, Wolk A (2007) Dietary carbohydrate, glycemic index, and glycemic load in relation to risk of colorectal cancer in women. Am J Epidemiol 165(3): 256-61

Levi F, Pasche C, Lucchini F, Bosetti C, La Vecchia C (2002) Glycaemic index, breast and colorectal cancer. Ann Oncol 13(10): 1688-9

Li HL, Yang G, Shu XO, Xiang YB, Chow WH, Ji BT, Zhang X, Cai H, Gao J, Gao YT, Zheng W (2011) Dietary glycemic load and risk of colorectal cancer in Chinese women. Am J Clin Nutr 93(1): 101-7

Makarem N, Bandera EV, Lin Y, Jacques PF, Hayes RB, Parekh N (2017) Carbohydrate nutrition and risk of adiposity-related cancers: results from the Framingham Offspring cohort (1991-2013). Br J Nutr 117(11): 1603-1614

McCarl M, Harnack L, Limburg PJ, Anderson KE, Folsom AR (2006) Incidence of colorectal cancer in relation to glycemic index and load in a cohort of women. Cancer Epidemiol Biomarkers Prev 15(5): 892-6

Michaud DS, Fuchs CS, Liu S, Willett WC, Colditz GA, Giovannucci E (2005) Dietary glycemic load, carbohydrate, sugar, and colorectal cancer risk in men and women. Cancer Epidemiol Biomarkers Prev 14(1): 138-47

Sieri S, Agnoli C, Pala V, Grioni S, Brighenti F, Pellegrini N, Masala G, Palli D, Mattiello A, Panico S, Ricceri F, Fasanelli F, Frasca G, Tumino R, Krogh V (2017) Dietary glycemic index, glycemic load, and cancer risk: results from the EPIC-Italy study. Sci Rep 7(1): 9757

Slattery ML, Benson J, Berry TD, Duncan D, Edwards SL, Caan BJ, Potter JD (1997) Dietary sugar and colon cancer. Cancer Epidemiol Biomarkers Prev 6(9): 677-85

Strayer L, Jacobs DR, Jr., Schairer C, Schatzkin A, Flood A (2007) Dietary carbohydrate, glycemic index, and glycemic load and the risk of colorectal cancer in the BCDDP cohort. Cancer Causes Control 18(8): 853-63

Terry PD, Jain M, Miller AB, Howe GR, Rohan TE (2003) Glycemic load, carbohydrate intake, and risk of colorectal cancer in women: a prospective cohort study. J Natl Cancer Inst 95(12): 914-6

Weijenberg MP, Mullie PF, Brants HA, Heinen MM, Goldbohm RA, van den Brandt PA (2008) Dietary glycemic load, glycemic index and colorectal cancer risk: results from the Netherlands Cohort Study. Int J Cancer 122(3): 620-9

Zelenskiy S, Thompson CL, Tucker TC, Li L (2014) High dietary glycemic load is associated with increased risk of colon cancer. Nutr Cancer 66(3): 362-8

2.3 Pancreas

George SM, Mayne ST, Leitzmann MF, Park Y, Schatzkin A, Flood A, Hollenbeck A, Subar AF (2009) Dietary glycemic index, glycemic load, and risk of cancer: a prospective cohort study. Am J Epidemiol 169(4): 462-72

Heinen MM, Verhage BA, Lumey L, Brants HA, Goldbohm RA, van den Brandt PA (2008) Glycemic load, glycemic index, and pancreatic cancer risk in the Netherlands Cohort Study. Am J Clin Nutr 87(4): 970-7

Hu J, La Vecchia C, Augustin LS, Negri E, de Groh M, Morrison H, Mery L (2013) Glycemic index, glycemic load and cancer risk. Ann Oncol 24(1): 245-51

Johnson KJ, Anderson KE, Harnack L, Hong CP, Folsom AR (2005) No association between dietary glycemic index or load and pancreatic cancer incidence in postmenopausal women. Cancer Epidemiol Biomarkers Prev 14(6): 1574-5

Meinhold CL, Dodd KW, Jiao L, Flood A, Shikany JM, Genkinger JM, Hayes RB, Stolzenberg-Solomon RZ (2010) Available carbohydrates, glycemic load, and pancreatic cancer: is there a link? Am J Epidemiol 171(11): 1174-82

Michaud DS, Liu S, Giovannucci E, Willett WC, Colditz GA, Fuchs CS (2002) Dietary sugar, glycemic load, and pancreatic cancer risk in a prospective study. J Natl Cancer Inst 94(17): 1293-300

Nothlings U, Murphy SP, Wilkens LR, Henderson BE, Kolonel LN (2007) Dietary glycemic load, added sugars, and carbohydrates as risk factors for pancreatic cancer: the Multiethnic Cohort Study. Am J Clin Nutr 86(5): 1495-501

Patel AV, McCullough ML, Pavluck AL, Jacobs EJ, Thun MJ, Calle EE (2007) Glycemic load, glycemic index, and carbohydrate intake in relation to pancreatic cancer risk in a large US cohort. Cancer Causes Control 18(3): 287-94

Rossi M, Lipworth L, Polesel J, Negri E, Bosetti C, Talamini R, McLaughlin JK, La Vecchia C (2010) Dietary glycemic index and glycemic load and risk of pancreatic cancer: a case-control study. Ann Epidemiol 20(6): 460-5

Sieri S, Agnoli C, Pala V, Grioni S, Brighenti F, Pellegrini N, Masala G, Palli D, Mattiello A, Panico S, Ricceri F, Fasanelli F, Frasca G, Tumino R, Krogh V (2017) Dietary glycemic index, glycemic load, and cancer risk: results from the EPIC-Italy study. Sci Rep 7(1): 9757

Silvera SA, Rohan TE, Jain M, Terry PD, Howe GR, Miller AB (2005) Glycemic index, glycemic load, and pancreatic cancer risk (Canada). Cancer Causes Control 16(4): 431-6

Simon MS, Shikany JM, Neuhouser ML, Rohan T, Nirmal K, Cui Y, Abrams J (2010) Glycemic index, glycemic load, and the risk of pancreatic cancer among postmenopausal women in the women's health initiative observational study and clinical trial. Cancer Causes Control 21(12): 2129-36

3. Other neoplasms

3.1 Lung

De Stefani E, Deneo-Pellegrini H, Mendilaharsu M, Ronco A, Carzoglio JC (1998) Dietary sugar and lung cancer: a case-control study in Uruguay. Nutr Cancer 31(2): 132-7

George SM, Mayne ST, Leitzmann MF, Park Y, Schatzkin A, Flood A, Hollenbeck A, Subar AF (2009) Dietary glycemic index, glycemic load, and risk of cancer: a prospective cohort study. Am J Epidemiol 169(4): 462-72

Hu J, La Vecchia C, Augustin LS, Negri E, de Groh M, Morrison H, Mery L (2013) Glycemic index, glycemic load and cancer risk. Ann Oncol 24(1): 245-51

Melkonian SC, Daniel CR, Ye Y, Pierzynski JA, Roth JA, Wu X (2016) Glycemic Index, Glycemic Load, and Lung Cancer Risk in Non-Hispanic Whites. Cancer Epidemiol Biomarkers Prev 25(3): 532-9

Sieri S, Agnoli C, Pala V, Grioni S, Brighenti F, Pellegrini N, Masala G, Palli D, Mattiello A, Panico S, Ricceri F, Fasanelli F, Frasca G, Tumino R, Krogh V (2017) Dietary glycemic index, glycemic load, and cancer risk: results from the EPIC-Italy study. Sci Rep 7(1): 9757

Sun JW, Zheng W, Li HL, Gao J, Yang G, Gao YT, Rothman N, Lan Q, Shu XO, Xiang YB (2018) Dietary Glycemic Load, Glycemic Index, and Carbohydrate Intake on the Risk of Lung Cancer among Men and Women in Shanghai. Nutr Cancer 70(4): 671-677

3.2 Bladder

Augustin LSA, Taborelli M, Montella M, Libra M, La Vecchia C, Tavani A, Crispo A, Grimaldi M, Facchini G, Jenkins DJA, Botti G, Serraino D, Polesel J (2017) Associations of dietary carbohydrates, glycaemic index and glycaemic load with risk of bladder cancer: a case-control study. Br J Nutr 118(9): 722-729

George SM, Mayne ST, Leitzmann MF, Park Y, Schatzkin A, Flood A, Hollenbeck A, Subar AF (2009) Dietary glycemic index, glycemic load, and risk of cancer: a prospective cohort study. Am J Epidemiol 169(4): 462-72

Hu J, La Vecchia C, Augustin LS, Negri E, de Groh M, Morrison H, Mery L (2013) Glycemic index, glycemic load and cancer risk. Ann Oncol 24(1): 245-51

Sieri S, Agnoli C, Pala V, Grioni S, Brighenti F, Pellegrini N, Masala G, Palli D, Mattiello A, Panico S, Ricceri F, Fasanelli F, Frasca G, Tumino R, Krogh V (2017) Dietary glycemic index, glycemic load, and cancer risk: results from the EPIC-Italy study. Sci Rep 7(1): 9757

3.3 Kidney

Galeone C, Pelucchi C, Maso LD, Negri E, Talamini R, Montella M, Ramazzotti V, Bellocco R, Franceschi S, La Vecchia C (2009) Glycemic index, glycemic load and renal cell carcinoma risk. Ann Oncol 20(11): 1881-5

George SM, Mayne ST, Leitzmann MF, Park Y, Schatzkin A, Flood A, Hollenbeck A, Subar AF (2009) Dietary glycemic index, glycemic load, and risk of cancer: a prospective cohort study. Am J Epidemiol 169(4): 462-72

Hu J, La Vecchia C, Augustin LS, Negri E, de Groh M, Morrison H, Mery L (2013) Glycemic index, glycemic load and cancer risk. Ann Oncol 24(1): 245-51

Sieri S, Agnoli C, Pala V, Grioni S, Brighenti F, Pellegrini N, Masala G, Palli D, Mattiello A, Panico S, Ricceri F, Fasanelli F, Frasca G, Tumino R, Krogh V (2017) Dietary glycemic index, glycemic load, and cancer risk: results from the EPIC-Italy study. Sci Rep 7(1): 9757

Zhu J, Tu H, Matin SF, Tannir NM, Wood CG, Wu X (2017) Glycemic index, glycemic load and carbohydrate intake in association with risk of renal cell carcinoma. Carcinogenesis 38(11): 1129-1135
